# MinD-like ATPase FlhG synchronizes flagellation and cell division in spirochetes

**DOI:** 10.1128/mbio.01834-26

**Published:** 2026-09-24

**Authors:** Kai Zhang, Wangbiao Guo, Michael J. Lynch, Brian R. Crane, Jun Liu, Chunhao Li

**Affiliations:** 1 Department of Oral Craniofacial Molecular Biology, Virginia Commonwealth University 6889, Richmond, Virginia, USA; 2 Department of Microbial Pathogenesis, Yale School of Medicine 5755 , New Haven, Connecticut, USA; 3 Microbial Sciences Institute, Yale University 5755, West Haven, Connecticut, USA; 4 Department of Chemistry and Chemical Biology, Cornell University 5922, Ithaca, New York, USA; Indiana University Bloomington, Bloomington, Indiana, USA

**Keywords:** spirochetes, cell division, flagellation, FlhG ATPase, spatial regulation

## Abstract

Spirochetes are evolutionarily distinct bacteria defined by their spiral morphology, unique means of motility, and periplasmic flagella (PFs). Because these flagella reside within the periplasm and are mechanically integrated with the cell body, their assembly must be precisely coordinated with cell growth and cytokinesis. However, the mechanism coupling flagellar biogenesis to cell division in spirochetes remains unclear. Using the Lyme disease spirochete *Borrelia burgdorferi* as a model, we identify FlhG (BB0269), a MinD-like ATPase, as a spatial regulator that links cell division to flagellar patterning. In wild-type cells, 7–11 long helical PFs form at each pole and form ribbon-like bundles that wrap around the cell cylinder to drive motility. Deletion of *flhG* disrupts this ordered architecture, resulting in pronounced heterogeneity in flagellar number, defective ribbon formation, aberrant septation, and severe motility defects. Mechanistically, FlhG dynamically localizes to the poles and division site, where it spatially organizes FlhF, an SRP-type GTPase that controls flagellar number and positioning, and FliF, the MS-ring protein that is an essential early component for flagellar biogenesis. Through this spatial regulation, FlhG coordinates flagellar biogenesis with cytokinetic progression. Together, these findings uncover a spatial regulatory mechanism that couples cell division to flagellation, providing insight into how spirochetes coordinate morphogenesis and motility to maintain their distinctive cellular architecture.

**IMPORTANCE:**

Spirochetes such as *Borrelia burgdorferi*, the causative agent of Lyme disease, rely on periplasmic flagella for motility and cell shape, yet how these structures are coordinated with cell division has remained unclear. We identify a MinD-like ATPase, FlhG, as a spatial regulator that couples flagellar assembly to cytokinesis. In contrast to its homologs in most bacteria, FlhG does not regulate flagellar protein levels but instead directs subcellular positioning of key assembly factors. By dynamically redistributing between the cell poles and division site, FlhG synchronizes flagellar patterning with septum formation. These findings uncover a previously unrecognized mechanism linking cell morphogenesis to the cell cycle and reveal how conserved ATPases can be repurposed to organize complex bacterial architectures.

## INTRODUCTION

Spirochetes represent a deeply branching lineage of bacteria distinguished by their spiral morphology and periplasmic flagella (PFs) (1–3). This phylum includes major human pathogens such as *Borrelia burgdorferi* (Lyme disease) (4), *Treponema pallidum* (syphilis) (5), and *Leptospira interrogans* (leptospirosis) (6). Unlike the external flagellate model organisms such as *Escherichia coli* and *Bacillus subtilis*, spirochetal PFs are confined within the periplasmic space, where they originate at both poles and extend inward along the cell cylinder (1). In most spirochetal species, these filaments assemble into ribbon-like bundles that wrap around the cell body, generating both the characteristic helical shape and the undulatory or corkscrew-like motility essential for environmental adaptation and host colonization (1, 2, 7–11). Thus, in spirochetes, flagella are structural determinants of cell morphology as well as engines of motility (1, 2, 12).

The integration of PFs within the periplasm imposes unique spatial constraints on their assembly (1, 2). During cell division, as new poles are generated, nascent PFs must be assembled and organized without disrupting preexisting flagellar bundles or cellular helicity. Consequently, spirochetes must synchronize flagellar biogenesis with cell division to preserve polarity, morphology, and motility. Failure to properly pattern these filaments disrupts ribbon architecture, alters cell shape, and compromises locomotion (1, 13–15). Although cryo-electron tomography (cryo-ET) and genetic analyses have provided substantial insight into the ultrastructure and hierarchical assembly of the spirochetal flagellar apparatus (7, 16, 17), the regulatory mechanisms that coordinate PFs assembly with division plane formation and polar morphogenesis remain poorly defined across the phylum.

ParA/MinD-family P-loop NTPases serve as key spatial regulators in many bacteria, orchestrating chromosome segregation, organelle positioning, and cytokinetic site selection (18–21). Among this family, FlhG, a MinD-like ATPase (also termed FleN in some species), has been extensively characterized in polar-flagellated bacteria, including *Vibrio* and *Campylobacter*, where it negatively regulates the number of polar flagella, and thus, deletion of *flhG* often leads to hyperflagellation (22–27). Through ATP-dependent dimerization and functional interactions with the SRP-like GTPase FlhF, FlhG coordinates flagellar biogenesis by regulating both flagellar assembly site selection and, in some species, flagellar gene expression (13, 24–26, 28). Whether analogous MinD-like spatial regulatory mechanisms operate in spirochetes, however, remains largely unknown. This question is particularly compelling because spirochetes possess periplasmic flagella, lack a canonical Min system (21, 29), and rely on these internal flagella not only for motility but also for maintaining their characteristic cell morphology (1, 12).

*Borrelia burgdorferi*, the causative agent of Lyme disease, provides a genetically tractable model for investigating the coordination of periplasmic flagellar (PF) assembly and cell cycle progression (2, 12, 29). This spirochete possesses an elongated protoplasmic cylinder, undergoes asynchronous chromosome replication, and exhibits noncanonical septation dynamics that differ fundamentally from those of rod-shaped model bacteria (30–32). The apparent absence of the canonical MinCDE division-site positioning system (21, 29, 33) suggests that spirochetes have evolved alternative mechanisms to coordinate polar morphogenesis, cytokinesis, and flagellar biogenesis. Similar to other polar flagellates, the genome of *B. burgdorferi* encodes a FlhG homolog (BB0269). Given its structural similarity to MinD-family ATPases and its established role in regulating polar flagellation in diverse bacterial species (22, 23, 26), FlhG is a compelling candidate for integrating these processes in *B. burgdorferi*.

Here, we demonstrate that the MinD-like ATPase FlhG coordinates spirochete cell division with flagellar assembly in *B. burgdorferi*. Using an integrated approach combining genetics, biochemistry, fluorescence microscopy, and cryo-ET, we show that FlhG regulates PFs' number and spatial organization while coordinating septation and polar morphogenesis. These findings identify FlhG as a key spatial regulator that links flagellar biogenesis to cytokinesis and provide a mechanistic framework for understanding how spirochetes coordinate morphogenesis with cell cycle progression.

## RESULTS

### BB0269 is a MinD-like ATPase

**Fig 1:**
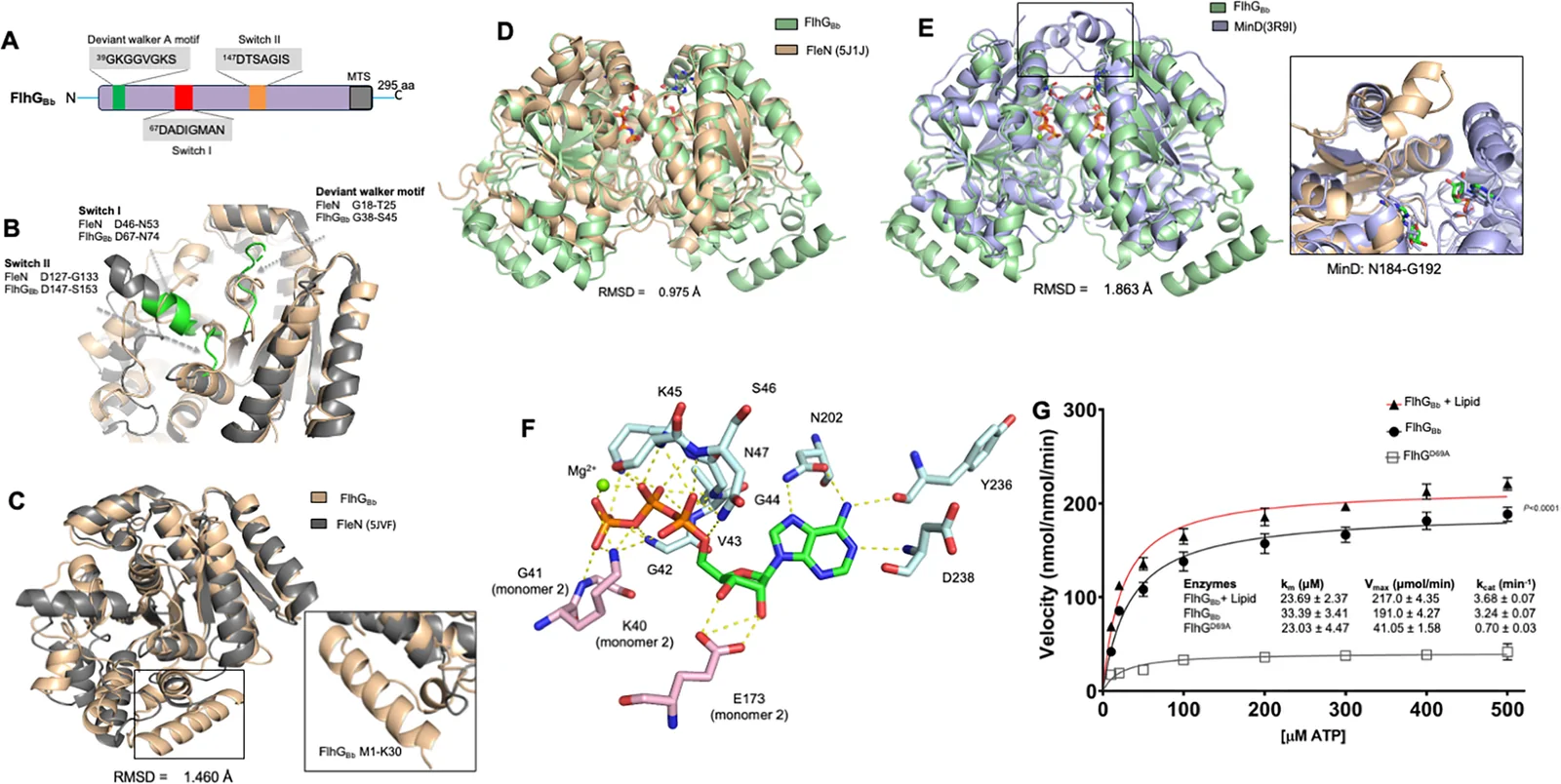
Structural and biochemical analyses of FlhG (FlhG_Bb_) from *B. burgdorferi*. (**A**) Schematic illustration of FlhG_Bb_ domain organization. The positions and amino acid sequence of the deviant Walker A, switch I, and switch II motifs are indicated. (**B**) Structural comparison of FlhG_Bb_ and *P. aeruginosa* FleN (PDB: 5JVF) monomers shows the deviant Walker A, switch I, and switch II motif labeled in green. (**C**) Structural superimposition of FlhG_Bb_ (tan) with FleN (PDB: 5JVF; dark gray) identifies a unique N-terminal α-helical extension (inset: M1-K30). (**D**) Structural superimposition of FlhG_Bb_ (green) with FleN (PDB: 5J1J; tan) dimers. RMSD = 0.975 Å. (**E**) Structural superimposition of the FlhG_Bb_ dimer (green) with the MinD dimer (PDB: 3R9I; blue). Inset shows a difference in the position of an interfacial helix comprising residues N184-G192. (**F**) Close-up view of the ATP-binding active site of FlhG_Bb_ showing key residues from both monomers involved in nucleotide coordination. (**G**) Michaelis-Menten kinetics of ATPase activity for wild-type FlhG_Bb_ (WT) with/without membrane lipid and D69 point mutant (D69A). Kinetic parameters (*K*_*m*_, *V*_max_, and *k*_cat_) are shown in the inset table. A representative result from three independent experiments with three biological replicates. Data represent mean ± SD.

BB0269 (295 amino acids; predicted molecular mass 32.77 kDa) is located within the *flgB* motility gene operon and is annotated as a FlhG-like ATP-binding protein (29, 34) (hereafter referred to as FlhG_Bb_). Sequence analysis revealed that FlhG_Bb_ belongs to the MinD/ParA family of P-loop NTPases (35, 36), sharing 25.73% and 32.58% sequence identity with *E. coli* MinD (37) and *Pseudomonas aeruginosa* FleN (a FlhG homolog) (38), respectively. Similar to other members of this family (35, 39), FlhG_Bb_ contains three conserved ATPase motifs: a deviant Walker A motif or P-loop (^39^GKGGVGKS⁴⁵), Switch I (⁶⁷DADIGMAN⁷⁴), Switch II (¹⁴⁷DTSAGIS¹⁵³), and a C-terminal membrane-targeting sequence (MTS) (40) (Fig. 1A; Fig. S1 and S2).

The AlphaFold structural model of FlhG_Bb_ closely resembles those of both MinD (RMSD = 1.863 Å; PDB: 3R9I [18]) and FleN (RMSD = 1.460 Å; PDB: 5JVF [41]), with overall greater structural similarity to FleN (Fig. 1B). Structural modeling further predicts that, unlike FleN, FlhG_Bb_ does not undergo a major conformational change upon ATP binding, either in the monomeric (Fig. S3) or dimeric states (Fig. S4). Another distinguishing feature is an N-terminal α-helical extension (residues M1-K30) that is absent from both MinD and FleN (Fig. 1C). Expanded sequence alignment revealed that this N-terminal extension is conserved in most FlhG homologs and contains the conserved D/NQAxxLR (Q4 in FlhG_Bb_) (Fig. S5). In *C. jejuni* and *V. alginolyticusthis*, this conserved glutamine is required for FlhG to function as a GTPase-activating protein (GAP) for FlhF, thereby coupling the activities of these two spatial regulators (24, 42, 43).

Structural superimposition also predicts that FlhG_Bb_ adopts a dimeric structure similar to that of *E. coli* MinD. However, the model reveals a distinct repositioning of an interfacial helix (residues N184-G192) at the predicted dimer interface (Fig. 1E), a configuration that more closely resembles that observed in FleN (Fig. 1D). Despite these structural variations, FlhG_Bb_ retains a conserved catalytic pocket predicted to coordinate ATP-Mg²^+^ binding (Fig. 1F).

To determine whether FlhG_Bb_ is enzymatically active, we performed *in vitro* ATPase assays using purified recombinant proteins. FlhG_Bb_ exhibited robust ATP hydrolysis activity following single-substrate steady-state kinetics (Fig. 1G). Substitution of Asp69 with alanine (D69A), a conserved residue required for ATP hydrolysis (22, 41), nearly abolished ATPase activity. In *E. coli*, binding of MinD MTS-associated phospholipids stimulates its ATPase activity (22, 40). To determine whether membrane lipids similarly regulate FlhG_Bb_, we measured ATP hydrolysis in the presence of *E. coli* polar lipids. The addition of polar lipid significantly (*P <* 0.0001) enhanced the ATPase activity of FlhG_Bb_ (Fig. 1G) suggesting that membrane association promotes its enzymatic function. Collectively, these results establish FlhG_Bb_ as a bona fide ATPase that shares the conserved biochemical properties of MinD/ParA family proteins despite subtle sequence and structural variations.

### Deletion of *flhG_Bb_* impairs *B. burgdorferi* growth and alters cell morphology

**Fig 2:**
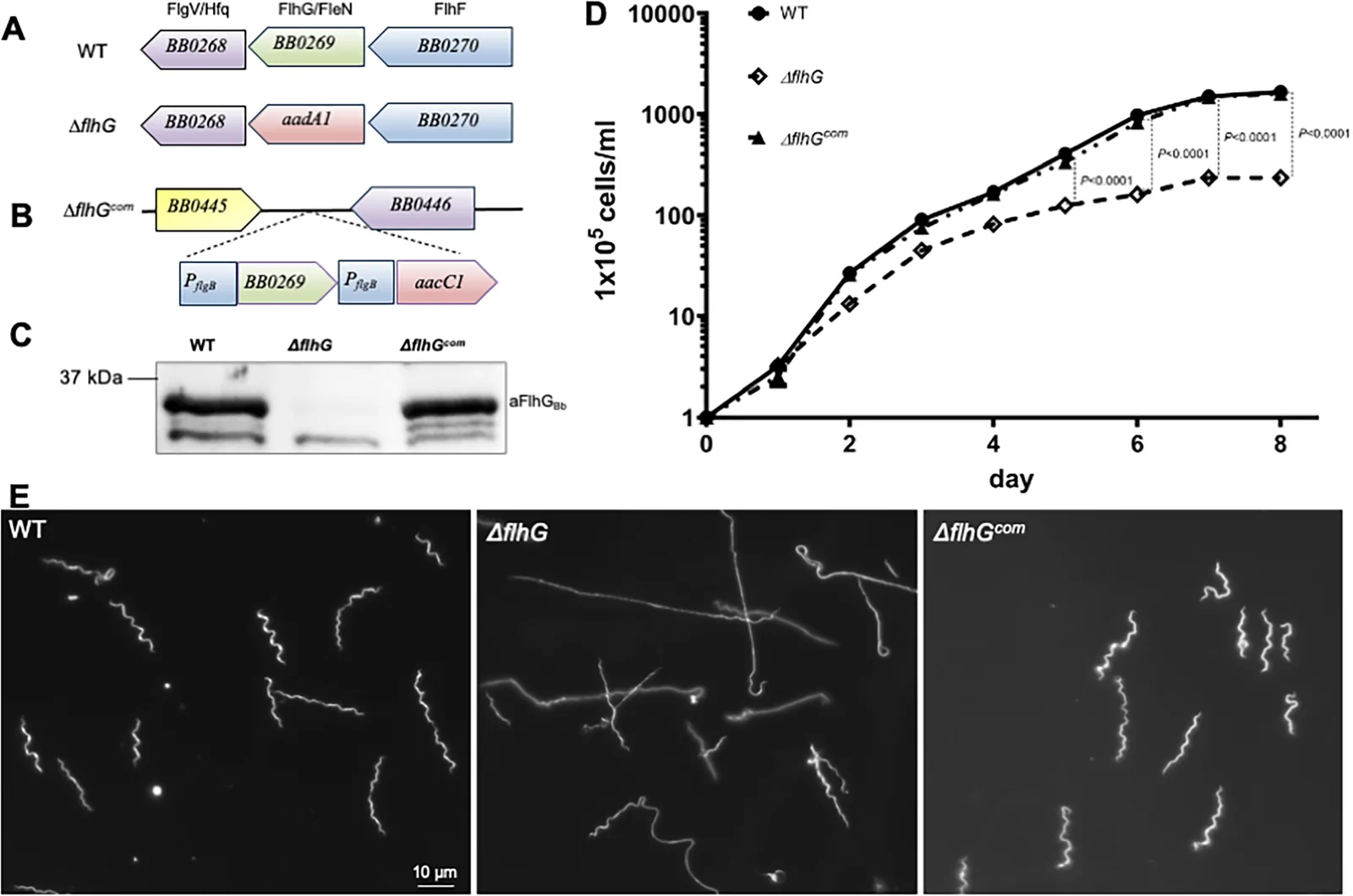
FlhG_Bb_ is required *for B. burgdorferi* normal growth and morphology. (**A**) Schematic of the chromosomal gene locus surrounding *flhG* (*BB0269*) in wild-type (WT) and the Δ*flhG* deletion mutant, in which *flhG* is in-frame replaced by the *aadA1* spectinomycin resistance cassette. (**B**) Schematic of cis-complementing the Δ*flhG* mutant by inserting *flgB-BB0269* into the intergenic region between BB0445 and BB0446, alongside a gentamicin resistance cassette (*aacC1*). (**C**) Immunoblot confirming FlhG_Bb_ expression in WT, Δ*flhG*, and Δ*flhG*^com^ strains probed with anti-FlhG_Bb_ antibody (αFlhG_Bb_). (**D**) Growth curves of WT, Δ*flhG*, and Δ*flhG*^com^ strains in BSK-II medium. Cell density was measured for up to 8 days using a Petroff–Hausser counting chamber. Statistical comparisons were performed at days 6, 7, and 8 (*P <* 0.0001). (**E**) Dark-field microscopy images of WT, Δ*flhG*, and Δ*flhG*^com^ strains. Images were taken at later-log growth phase. Scale bar = 10 µm.

The *flhG_Bb_* gene is located between *flhF* (*BB0270*) (13) and *flgV* (*BB0268*) (44, 45) in the *flgB* motility gene operon (34). To minimize potential polar effects on the expression of adjacent genes, *flhG_Bb_* (*BB0269*) was deleted and replaced in-frame with a streptomycin-resistance cassette (*aadA1*) (Fig. 2A). The resulting mutant (Δ*flhG*) was complemented by inserting the *flgB-BB0269* or *flgB-BB0269* with a point mutation in D69A fragment into the intergenic region between *BB0445* and *BB0446*, as previously described (46) (Fig. 2B). The resulting two complemented strains were designated as Δ*flhG*^com^ and Δ*flhG*^D69A^. Immunoblot analysis confirmed that FlhG_Bb_ was absent in the Δ*flhG* mutant and restored in the complemented strain Δ*flhG*^com^ (Fig. 2C). The Δ*flhG* mutant exhibited a pronounced growth defect (Fig. 2D). At stationary phase, the mutant reached a cell density of 2.34 × 10⁷ cells mL⁻¹, representing an approximately sevenfold reduction compared with the wild type (1.59 × 10⁸ cells mL⁻¹). This growth defect was rescued by complementation.

Dark-field microscopy further revealed striking morphological abnormalities in mutant cells. Wild-type cells displayed the characteristic wave-like morphology and were relatively uniform in length. In contrast, most Δ*flhG* cells adopted a rod-like morphology and were more diverse in terms of their sizes (Fig. 2E). Detailed measurements revealed two predominant mutant populations: elongated rod-shaped cells (>70% of cells; average length 39.41 ± 13.33 µm, *n* = 22) and short spiral cells (<30%; average length 11.53 ± 3.48 µm, *n* = 16). The elongated rods were approximately twice the length of wild-type cells (17.27 ± 3.39 µm, *n* = 37), whereas the short cells were roughly half the wild-type length. These results indicate that loss of FlhG_Bb_ disrupts normal cell division and morphogenesis in *B. burgdorferi*.

### FlhG_Bb_ is essential for the motility of *B. burgdorferi*

**Fig 3:**
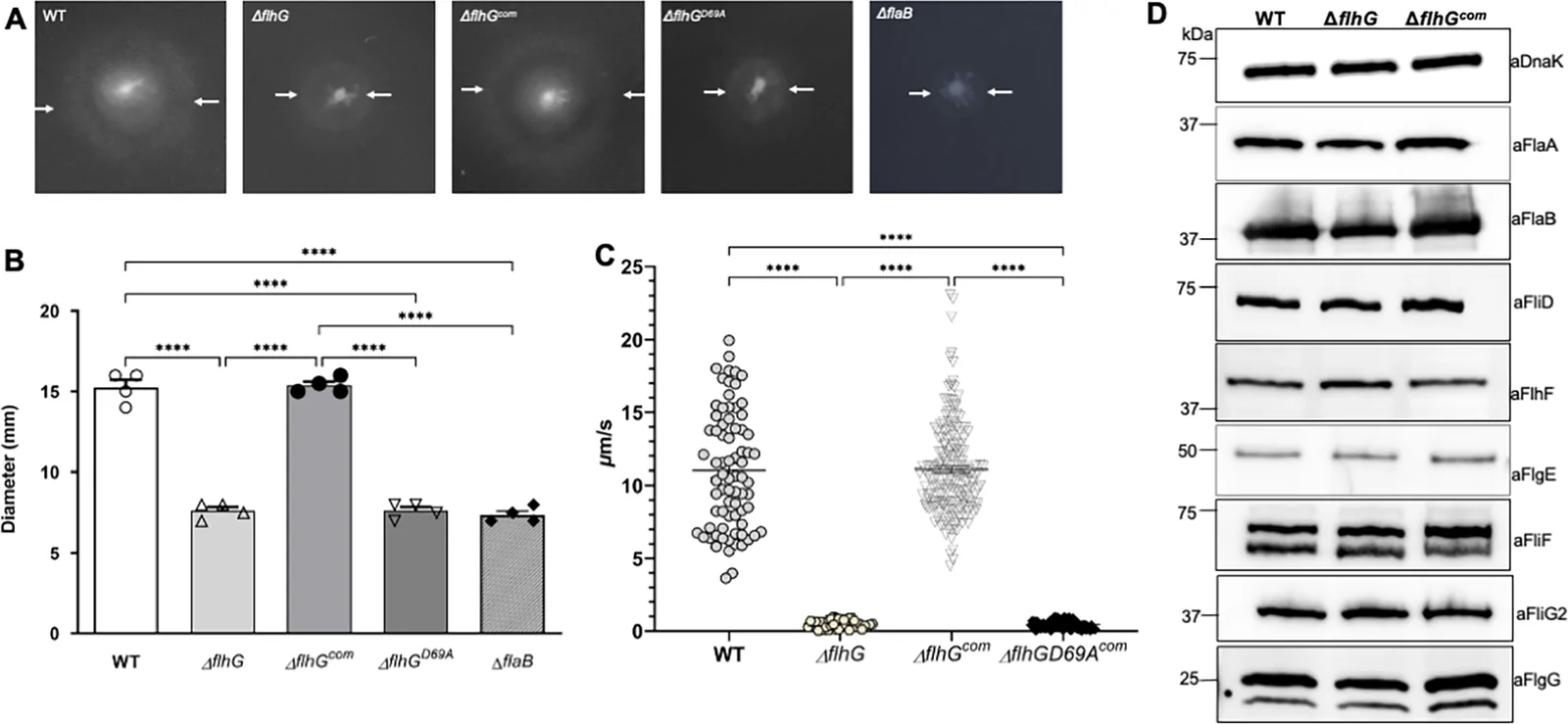
FlhG ATPase activity is required for motility but not flagellar protein synthesis. (**A**) Simming plate assays of WT, Δ*flhG*, Δ*flhG*^com^, and Δ*flhG*^D69A^ strains. Δ*flaB*, a non-motile mutant (12), was used as a control to measure the inoculum size. Arrow pairs indicate the outer border of swimming rings. (**B**) Quantification of swimming ring diameters (mm) for the indicated strains. Data shown as mean ± SD with individual data points. ****P < 0.0001*.* (**C**) Swimming speeds (μm/s) of individual cells for WT, Δ*flhG*, Δ*flhG*^com^, and Δ*flhG^D69A^* strains measured by bacterial motion tracking analysis. ****P < 0.0001. (**D**) Immunoblot analysis of flagellar and motor structural proteins in WT, Δ*flhG*, and Δ*flhG*^com^ strains. Whole-cell lysates were probed with antibodies against DnaK (loading control), FlaA, FlaB, FliD, FlhF, FlgE, FliF, FliG2, and FlgG as indicated. Molecular weight markers (kDa) are shown.

To assess the role of FlhG_Bb_ in motility, we performed swimming plate assays in 0.3% semisolid agar. The Δ*flhG* mutant produced significantly smaller migration rings than the wild type (WT: ~15.2 mm vs Δ*flhG*: ~7.9 mm; *P* < 0.0001) (Fig. 3A and B). Complementation with wild-type *flhG_Bb_* fully restored the migration ring diameter (~15.2 mm). In contrast, complementation with an ATPase-deficient point mutant (Δ*flhG^D69A^*) failed to rescue motility (~7.6 mm; *P* < 0.0001 vs WT) (Fig. 3B). These results indicate that the ATPase activity of FlhG_Bb_ is required for normal motility. Consistent with the swimming plate assays, quantitative tracking analysis revealed that wild-type and complemented (Δ*flhG*^com^) cells displayed robust translational movement (Videos S1 and S3) and their averaged velocity in 1% methylcellulose reached ~11 µm/s (WT: 75 cells; Δ*flhG*^com^: 96 cells), whereas Δ*flhG* and Δ*flhG^D69A^* cells exhibited wave-like body undulation (Videos S2 and S4) but failed to move (~0 µm/s; Δ*flhG*: 47 cells; Δ*flhG^D69A^:* 71 cells) (Fig. 3C). Together, these findings demonstrate that FlhG_Bb_ and its ATPase activity are essential for *B. burgdorferi* motility.

### FlhG_Bb_ does not regulate flagellar protein synthesis

FlhG has been functionally characterized in several polar flagellates (25, 35). In some bacteria, including *Vibrio cholerae* and *P. aeruginosa*, deletion of *flhG* leads to elevated levels of flagellar proteins accompanied by hyperflagellation (47, 48). In contrast, deletion *of flhG* in *C. jejuni* and *Helicobacter pylori* causes defects in flagellar number and positioning without significantly affecting flagellar protein abundance (24, 28). To determine whether FlhG_Bb_ controls flagellar protein synthesis, we compared the abundance of several representative flagellar proteins in wild-type and Δ*flhG* strains by immunoblotting. The proteins examined included the MS-ring protein FliF (16), the motor switch protein FliG2 (8), the major flagellin FlaB (12), the flagellar sheath protein FlaA (49), the filament cap protein FliD (50), and the SRP-like GTPase FlhF (13). All proteins were present at comparable levels in the two strains (Fig. 3D), indicating that loss of FlhG_Bb_ does not alter the abundance of these major flagellar components. These findings place *B. burgdorferi* alongside *C. jejuni* and *H. pylori*, in which FlhG primarily regulates flagellar number and spatial organization rather than flagellar protein production.

### Loss of FlhG_Bb_ leads to aberrant flagellar patterning

**Fig 4:**
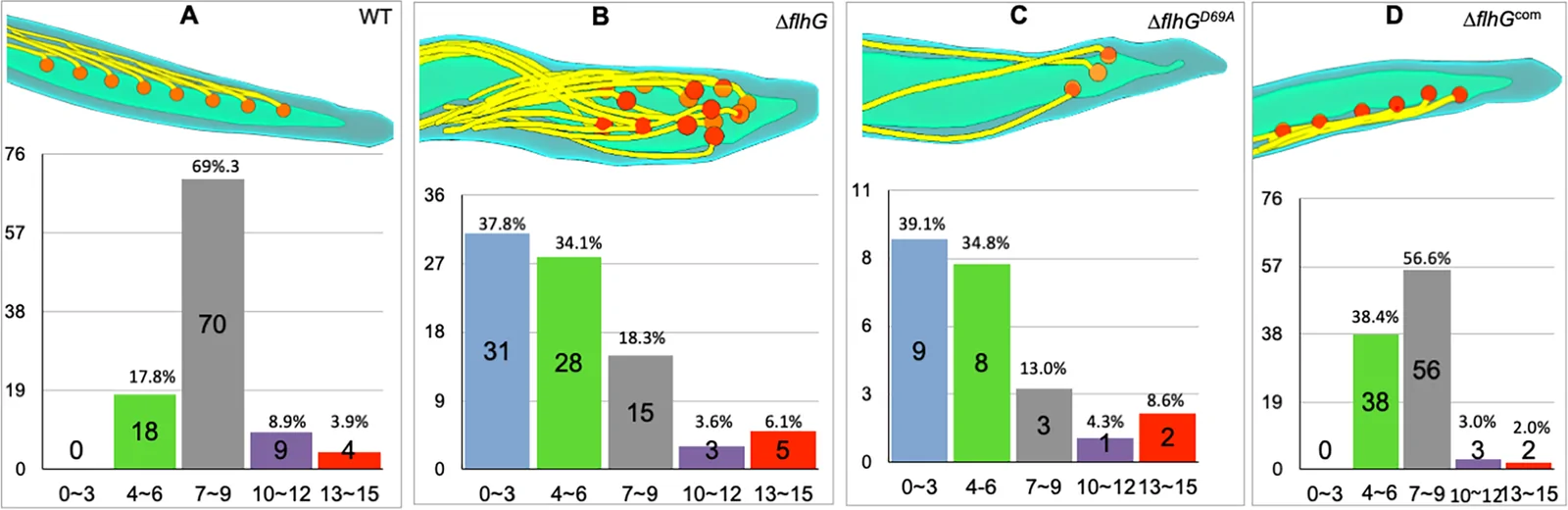
Loss of *flhG_Bb_* results in dysregulation of flagellar motor number. (**A–D**) Top row: representative segmentation of a cell tip illustrating flagellar distributions in wild type (WT) (**A**), Δ*flhG* (**B**), Δ*flhG*^D69A^ (**C**), and Δ*flhG*^com^ (**D**). Bottom row: histograms showing the distribution of flagellar numbers per cell tip for WT (**A**), Δ*flhG* (**B**), Δ*flhG*^D69A^ (**C**), and Δ*flhG*^com^ (**D**). Numbers inside bars indicate cell tip counts.

FlhG_Bb_ is essential for *B. burgdorferi* motility; however, its deletion does not significantly affect flagellar protein synthesis, leaving the mechanistic basis of the motility defect unclear. To address this, we examined flagellar architecture in Δ*flhG* and complemented strains by cryo-ET. *B. burgdorferi* cells typically have 7–11 PFs per pole (Fig. 4A), which extend inward and form two ribbon-like bundles that tightly wrap around the cell cylinder (7, 13, 17). In Δ*flhG* cells, however, this pattern was severely disrupted: PF numbers were highly variable, ranging from 2 to 15 per cell pole, and flagellar motors were distributed without discernible pattern, failing to coalesce into ribbon-like structures (Fig. 4B). A comparable disorganization was observed in the D69A point mutant (Fig. 4C), indicating that the regulatory function of FlhG_Bb_ on flagellar patterning depends on its ATPase activity. In contrast, complemented cells fully restored the wild-type flagellar pattern, with 7–11 PFs forming characteristic tight ribbon bundles that wrap around the cell body and extend toward the cell center (Fig. 4A and D). Collectively, these findings establish that FlhG_Bb_ governs both flagellar number and spatial organization, two properties that are essential for coordinated motility in *B. burgdorferi*.

### Loss of FlhG_Bb_ disrupts the flagellar motor collar structure

**Fig 5:**
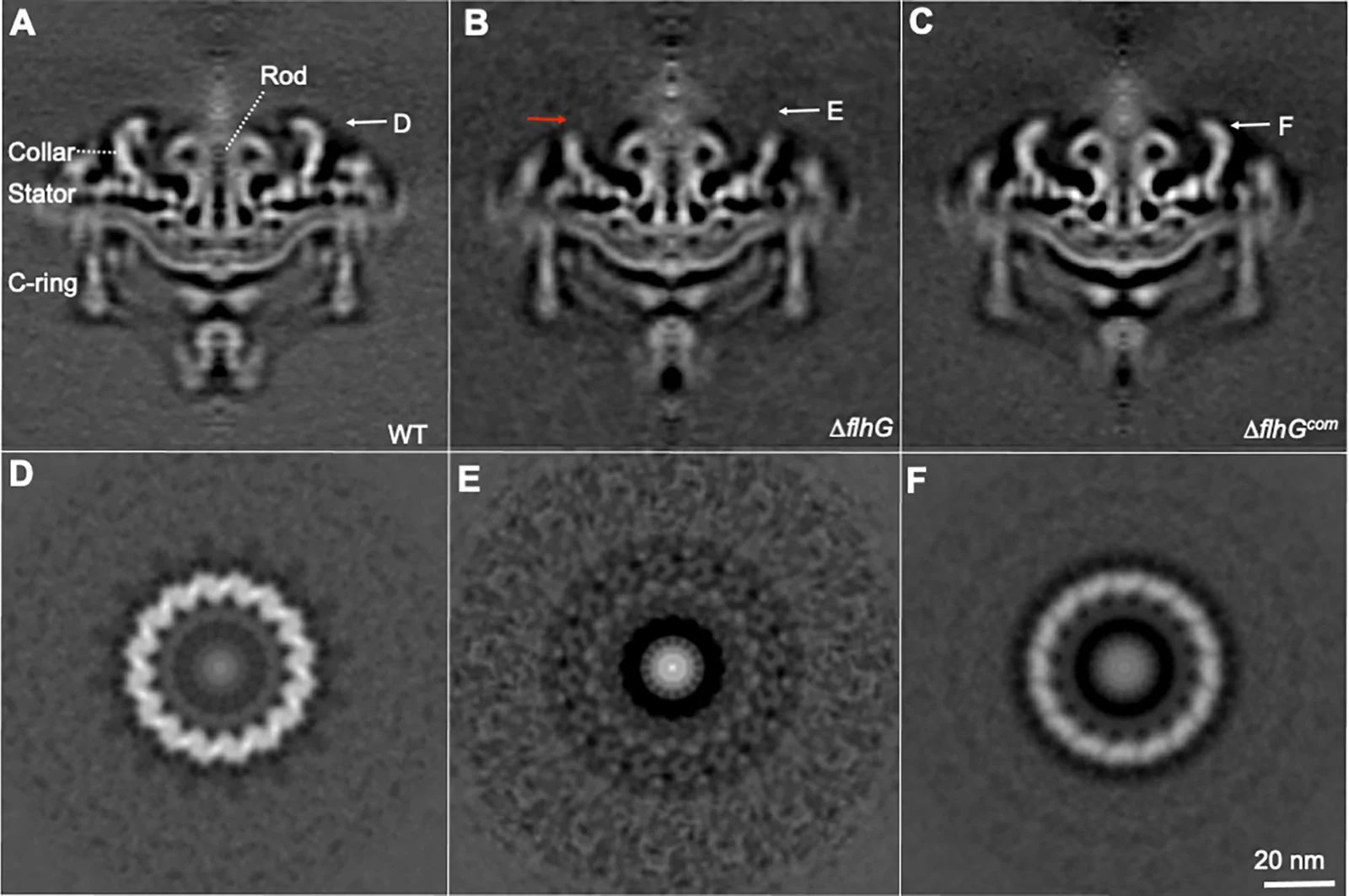
FlhG_Bb_ is required for proper flagellar collar assembly as revealed by cryo-ET. (**A–C**) Longitudinal cross-sectional views of subtomogram averages of the flagellar motor from WT (**A**), Δ*flhG* (**B**), and Δ*flhG*^com^ (**C**). Arrows indicate the positions of the disk (**D–F**) structures referenced in panels **A–C**. (**D–F**) Top-down cross-sectional views at the outer disk for WT (**D**), Δ*flhG* (**E**), and Δ*flhG*^com^ (**F**). Red arrows indicate the disk density. Deletion of *flhG_Bb_* results in loss of the characteristic disk density (**E**), which is restored upon complementation (**F**).

In addition to flagellar patterning, we also examined the potential impact of FlhG_Bb_ on the ultrastructure of flagellar motors *in situ* using cryo-ET and subtomogram averaging. Strikingly, subtomogram averaging revealed that a ring-like structure present in the wild-type flagellar motor (Fig. 5A and D) was absent in the Δ*flhG* mutant (Fig. 5B and E). Importantly, this structural defect was rescued in the complemented strain (Fig. 5C and F). This ring-like structure sits atop the flagellar collar, a hallmark feature unique to spirochetal flagellar motors (51–53), suggesting that FlhG_Bb_ plays a role in motor architecture. Collectively, these findings establish FlhG_Bb_ as a critical determinant of collar integrity, though the precise molecular mechanism by which it governs flagellar collar assembly remains to be determined.

### Deletion of *flhG_Bb_* leads to aberrant cell division

**Fig 6:**
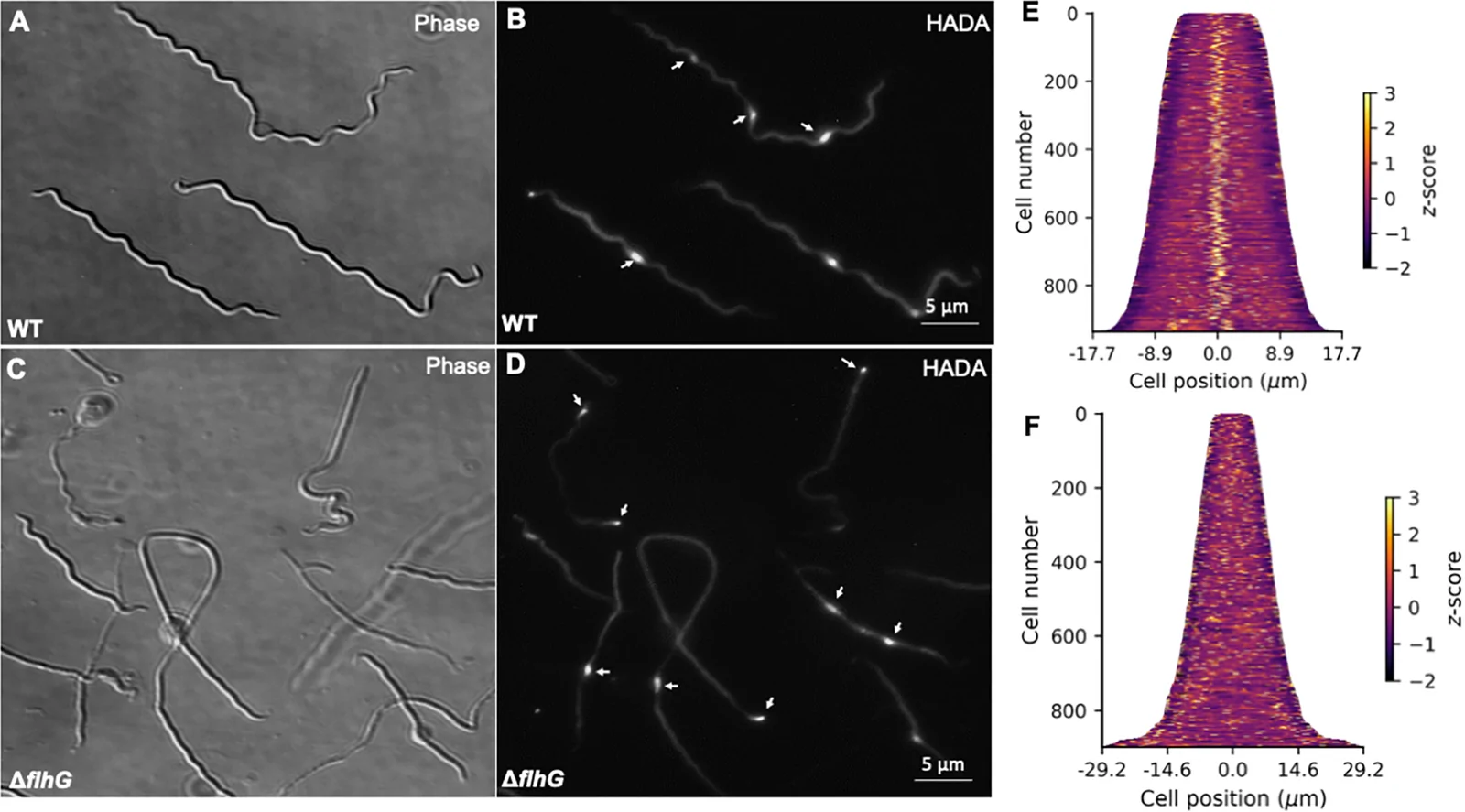
Loss of flhG_Bb_ leads to aberrant cell division in *B. burgdorferi*. (**A and C**) Phase contrast (left) and HADA fluorescence (right) images of WT (**A**) and Δ*flhG* (**C**) cells labeled with the fluorescent D-amino acid HADA to visualize sites of active peptidoglycan synthesis. White arrows indicate HADA incorporation foci. Scale bar = 5 µm. (**B and D**) Heat maps of normalized HADA fluorescence intensity as a function of cell body position (μm) for WT (**B**) and Δ*flhG* (**D**). Each row represents a single cell (*n* ≥ 900 cells per strain), with *z*-score-normalized fluorescence depicted on a color scale (purple to yellow). Δ*flhG* cells show a wider distribution of cell lengths compared to WT.

Deletion of *flhG_Bb_* affects *B. burgdorferi* growth and cell morphology (Fig. 2), suggesting a potential role in cell division. To test this possibility, we employed HADA labeling (54) to visualize newly synthesized peptidoglycan in live cells and monitor septum formation. Consistent with a previous report (30), wild-type cells exhibited discrete HADA signals at division sites that followed the characteristic 1/4, 1/2, and 3/4 pattern (Fig. 6A). Demographic analysis further showed that most wild-type cells displayed a single HADA band at midcell (Fig. 6B; *n* = 936 cells). In contrast, this spatial pattern was markedly disrupted in Δ*flhG* cells (Fig. 6C and D; *n* = 900 cells). Specifically, 66.7% ± 7.3% of mutant cells exhibited irregular high-intensity HADA signals that did not conform to the 1/4, 1/2, and 3/4 rule. In addition, 20.9% ± 6.5% of cells displayed strong signals at one cell end, 7.4% ± 3.8% at midcell, and 4.9% ± 3.8% at both cell ends. These aberrant labeling patterns indicate that septum positioning and peptidoglycan synthesis become spatially misregulated in the absence of FlhG. Together, these findings demonstrate that FlhG_Bb_ contributes to proper division-site placement in *B. burgdorferi*, supporting a role for this ATPase in coordinating cell division with flagellar organization.

### FlhG_Bb_ localizes to the cell poles and midcell

**Fig 7:**
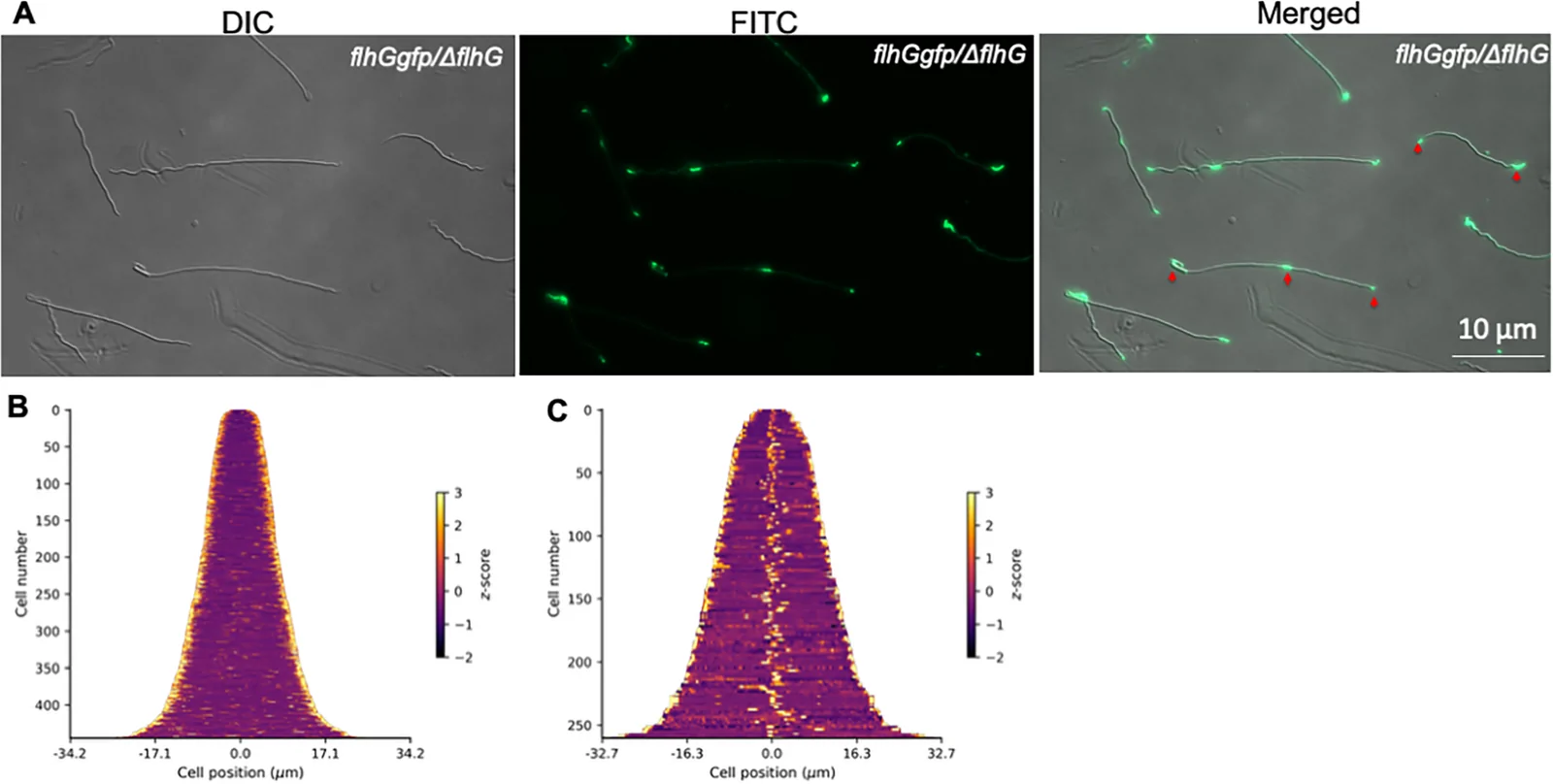
FlhG-GFP localizes to the flagellar insertion points in *B. burgdorferi*. (**A**) Fluorescence microscopy of *flhGgfp/*Δ*flhG* cells expressing FlhG-GFP as the sole copy of FlhG. DIC (left), FITC fluorescence (middle), and merged (right) channels are shown. FlhG-GFP signal appears as discrete foci distributed along the cell body, consistent with flagellar insertion sites. Scale bar = 10 µm. (**B**) Demographic distribution of FlhG-GFP in newborn cells (*n* = 445 cells). (**C**) Demographic distribution of FlhG-GFP in dividing cells (*n* = 269 cells).

To determine how FlhG_Bb_ regulates flagellar patterning and cell division, we examined its subcellular localization using a GFP fusion approach. A plasmid expressing FlhG_Bb_-GFP was constructed and introduced into the *flhG* mutant strain. Immunoblot analysis with antibodies against GFP and FlhG_Bb_ confirmed expression of the fusion protein. Fluorescence microscopy combined with demographic analysis revealed that GFP puncta were predominantly detected at the cell poles in short newborn cells, whereas in longer dividing cells fluorescence signals were observed at both poles and the midcell division site (Fig. 7A through C). A similar localization pattern was observed when the FlhG_Bb_-GFP expression vector was introduced into wild-type cells (Fig. S6). Together, these results indicate that FlhG_Bb_ exhibits dynamic localization, residing primarily at the cell poles in nascent cells and redistributing to the midcell division zone during cell elongation and division. This dynamic, cell-cycle-dependent localization, reminiscent of MinD in rod-shaped bacteria (19, 21), likely directs proper flagellar assembly and synchronizes it with cytokinesis in *B. burgdorferi*.

### FlhG_Bb_ controls the polar localization of FlhF and FliF

FlhF is a key regulator of both the number and positioning of PFs in *B. burgdorferi* and is essential for maintaining the flat-ribbon flagellar architecture (13). Its function involves stabilizing critical flagellar components, including the MS-ring protein FliF, a central structural element of the flagellar motor (16, 55). We, therefore, hypothesized that FlhG_Bb_ regulates flagellar patterning through FlhF and FliF. To test this hypothesis, we expressed FlhF-GFP and FliF-GFP in their respective deletion mutants (*ΔflhF* and Δ*fliF*) as well as in the Δ*flhG* mutant and then compared their subcellular localization by fluorescence microcopy.

**Fig 8:**
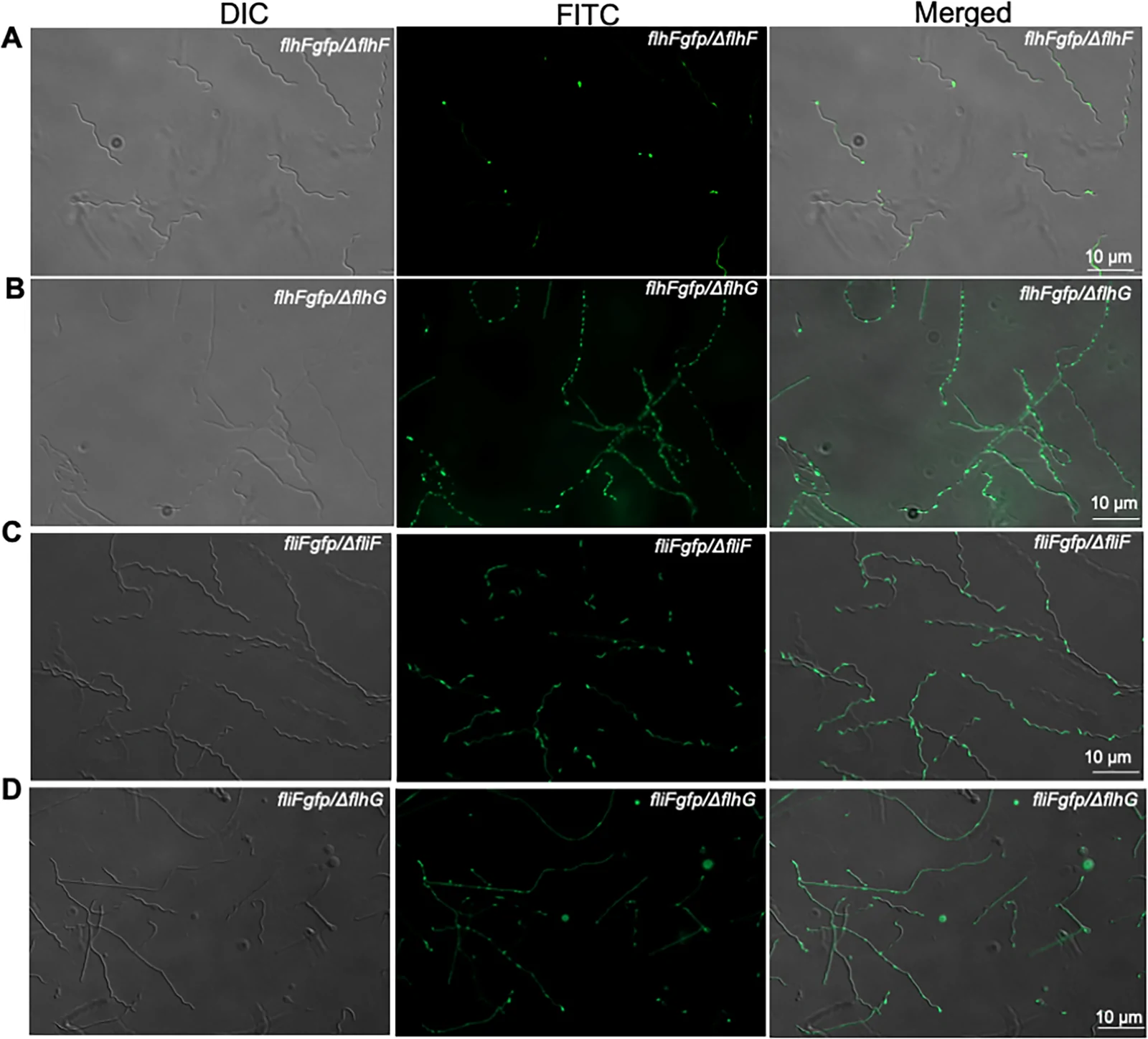
FlhGBb is required for the polar localization of FlhF and FliF. (**A**) Fluorescence microscopy of *flhFgfp/*Δ*flhF* cells expressing FlhF-GFP in a Δ*flhF* background. DIC (left), FITC fluorescence (middle), and merged (right) channels are shown. FlhF-GFP localizes as discrete polar or subpolar foci, consistent with flagellar tip/insertion localization. Scale bar = 10 µm. (**B**) Fluorescence microscopy of *flhFgfp/*Δ*flhG* cells expressing FlhF-GFP in a Δ*flhG* background. In the absence of FlhG, FlhF-GFP signal is redistributed along the cell body as multiple, evenly spaced foci rather than discrete polar clusters, consistent with derepressed flagellar assembly at ectopic sites. Scale bar = 10 µm. (**C**) Fluorescence microscopy of *fliFgfp*/B31A WT cells expressing FliF-GFP. DIC (left), FITC fluorescence (middle), and merged (right) channels are shown. FliF-GFP localizes as discrete foci at the cell poles, consistent with a limited number of polar flagellar motors per cell. Scale bar = 10 µm. (**D**) Fluorescence microscopy of *fliFgfp*/Δ*flhG* cells. In the absence of FlhG_Bb_, FliF-GFP foci are no longer restricted to the cell poles but are instead distributed along the entire cell length, indicating uncontrolled flagellar MS-ring assembly at ectopic sites. Scale bar = 10 µm.

In the Δ*flhF* strain, FlhF-GFP localized predominantly to the cell poles, with an average of two polar foci per cell (*n* = 22) (Fig. 8A). In contrast, deletion of *flhG* completely disrupted the polar localization of FlhF, resulting in numerous dispersed FlhF-GFP puncta distributed throughout the cell (18.93 ± 5.43 puncta per cell, *n* = 29) (Fig. 8B). Likewise, FliF-GFP formed discrete polar foci in the Δ*fliF* strain (2.89 ± 0.47 puncta per cell, *n* = 36) (Fig. 8C), whereas in the Δ*flhG* mutant, it became broadly distributed along the cell length, with a significantly increased number of fluorescence puncta (8.96 ± 3.49 per cell, *n* = 29) (Fig. 8D). These findings demonstrate that FlhG_Bb_ is required for the polar targeting of both FlhF and FliF, thereby coordinating flagellar assembly, number, and spatial organization.

## DISCUSSION

In most rod-shaped bacteria, accurate cell division is governed by the MinCDE system, in which the ATPase MinD oscillates between the cell poles to recruit the division inhibitor MinC, thereby preventing polar FtsZ assembly and restricting Z-ring formation to midcell (56–59). In contrast, *B. burgdorferi* lacks the canonical MinCDE system (29, 33), indicating that it has evolved a fundamentally different mechanism for division-site selection. In this spirochete, cytokinesis must be tightly coordinated with chromosome segregation, polar morphogenesis, and periplasmic flagellar assembly, which are essential not only for motility but also for maintaining the characteristic flat-wave cell morphology (13, 30–32). However, the molecular machinery that spatially coordinates these processes has remained largely unknown.

Although FlhG_Bb_ shares only ~26% sequence identity with *E. coli* MinD, it retains the structural features of MinD-family ATPases, including a deviant Walker A (P-loop) motif, Switch I and II regions, and a C-terminal membrane-targeting sequence (MTS) (Fig. 1). FlhG_Bb_ also contains the invariant catalytic residue Asp69, which is essential for ATP hydrolysis, and its ATPase activity is stimulated by membrane-associated lipids, a hallmark of MinD function (Fig. 1G). Despite these conserved biochemical properties, loss of FlhG_Bb_ results in profound defects in cell morphology, motility (Fig. 3), flagellar patterning (Fig. 4), and cytokinesis (Fig. 6), indicating that its cellular function has diverged from that of canonical MinD. We, therefore, propose that FlhG_Bb_ has repurposed the conserved MinD ATPase scaffold to serve as a spirochete-specific spatial organizer that coordinates cytokinesis with polar flagellar assembly, thereby ensuring precise cell division and inheritance of the motility machinery.

A central insight from this work is that FlhG_Bb_ regulates flagellar biogenesis primarily through spatial organization rather than changes in flagellar protein abundance (Fig. 3D). A similar mode of regulation has been reported in *C. jejuni*, where FlhG does not control flagellar number by altering flagellar gene expression or protein production (24, 26). In contrast, in many rod-shaped bacteria, FlhG homologs regulate flagellar number through transcriptional or post-translational pathways. For example, in *Vibrio* species, FlhG interacts with the transcriptional activator FlrA to repress flagellar gene expression and thereby limit polar flagellation (60). Similarly, the *Pseudomonas* FlhG homolog FleN controls flagellar number by modulating transcriptional regulators of the flagellar gene hierarchy (27, 61). In spirochetes, deletion of *flhG* in *L. biflexa* has no impact on flagellar gene expression but instead alters the global transcriptome (14). Collectively, these observations suggest that although the role of FlhG homologs in regulating flagellation is broadly conserved, the underlying regulatory mechanisms vary across bacterial species.

Consistent with this concept, our findings indicate that FlhG_Bb_ acts primarily at the level of subcellular organization, directing the localization of key flagellar assembly factors. Specifically, FlhG_Bb_ controls the positioning of FlhF (Fig. 8), an SRP-type GTPase that specifies flagellar placement (13), and FliF, the MS-ring protein that is essential for basal body assembly (16, 62). Correct localization of these components ensures that flagellar basal bodies assemble at defined polar sites and generate the ribbon-like flagellar bundles characteristic of spirochetes. When FlhG_Bb_ is absent, both FlhF and FliF lose polar confinement and appear as dispersed puncta along the cell body (Fig. 8), indicating that spatial control of early assembly events is critical for establishing the ordered architecture of the spirochete flagella. This regulatory mode resembles aspects of FlhG function described in *C. jejuni*, where FlhG influences polar flagellar numbers (24, 26, 63). However, the extensive spatial control observed here may be particularly important in spirochetes, whose PFs are mechanically integrated with the cell cylinder.

**Fig 9:**
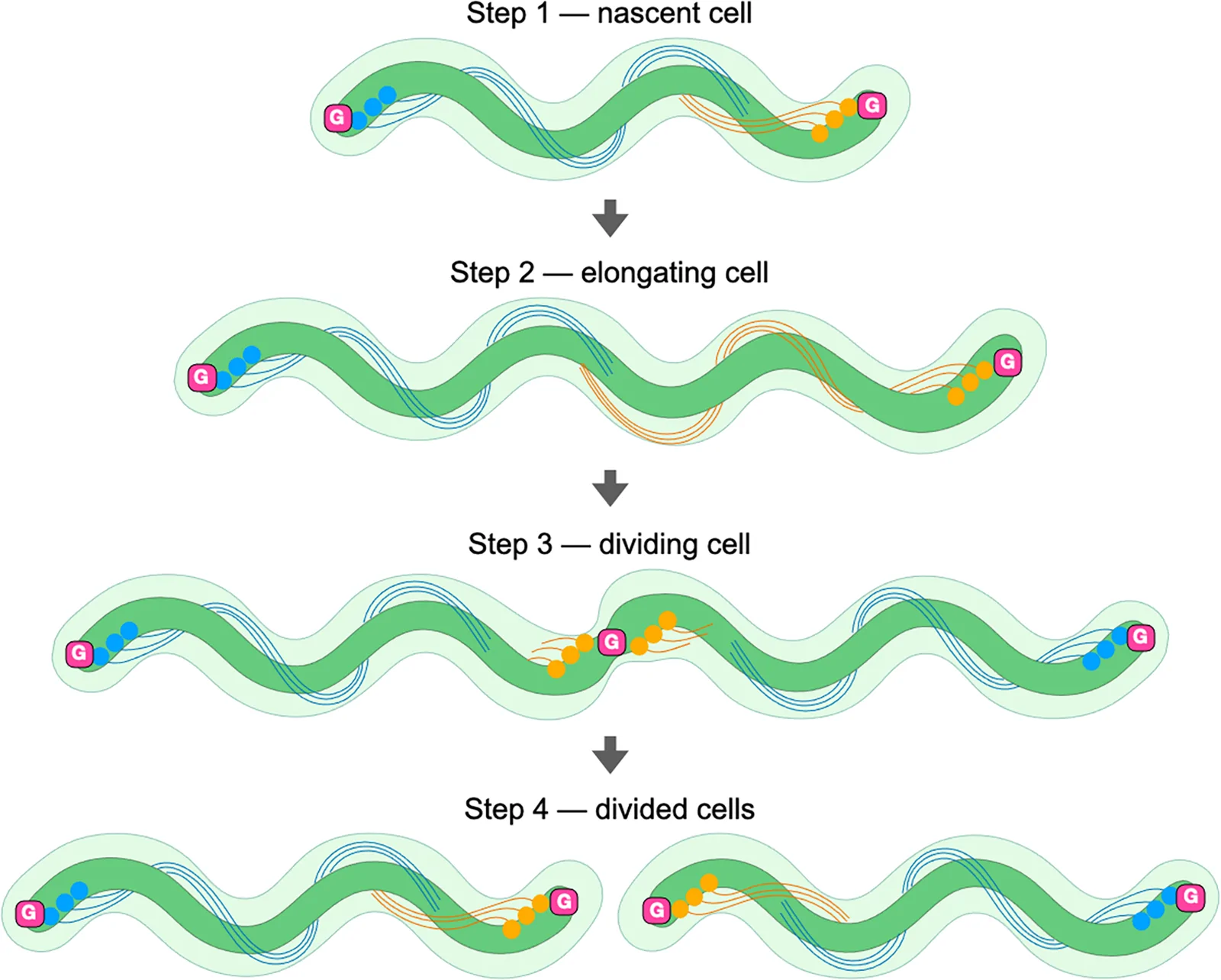
Model for FlhG_Bb_-mediated coordination of flagellation and cell division in *B. burgdorferi*. In nascent cells, the ATPase FlhG_Bb_ localizes predominantly at the cell poles, where it recruits and spatially organizes FlhF and the MS-ring protein FliF to establish sites of basal body assembly. These events initiate the formation of polar PFs that assemble into ribbon-like bundles along the cell cylinder. As the cell cycle progresses, FlhG_Bb_ redistributes toward the midcell division zone, where it likely contributes to proper septum placement and coordination of cytokinesis with ongoing flagellar morphogenesis. In the absence of FlhG_Bb_, FlhF and FliF become delocalized, basal bodies assemble at aberrant positions, and organized PF ribbon formation is disrupted, leading to defects in motility, cell morphology, and division. Through this ATPase-dependent spatial regulation, FlhG_Bb_ synchronizes flagellar assembly with cell division to maintain the distinctive architecture and motility of spirochetes.

Beyond regulating flagellar number, FlhG_Bb_ also appears to coordinate flagellar biogenesis with the cell division cycle. FlhG_Bb_ exhibits dynamic localization, concentrating at cell poles in newly formed cells and redistributing toward the division site during septation (Fig. 7). This behavior resembles the spatial dynamics of MinD in rod-shaped bacteria (18, 57–59) and suggests that FlhG_Bb_ may provide positional information that couples polar development with cytokinesis. Based on these findings, we propose a mechanistic model (Fig. 9) in which FlhG_Bb_ acts as a master spatial organizer in *B. burgdorferi*. At the poles, FlhG_Bb_ directs the localization of FlhF and FliF to establish sites of basal body assembly and promote formation of organized PF ribbon bundles. As cells progress through the division cycle, redistribution of FlhG_Bb_ toward midcell likely contributes to proper septum placement, synchronizing cytokinesis with ongoing flagellar assembly. Through this ATPase-driven localization cycle, FlhG_Bb_ integrates morphogenesis, motility, and cell division to maintain the distinctive spiral morphology and highly ordered flagellar architecture of spirochetes.

FlhG_Bb_ may also influence higher-order flagellar motor architecture. Cryo-ET revealed that the ring-like structure associated with the flagellar collar is absent in Δ*flhG* cells (Fig. 5). The collar is a distinctive feature that stabilizes the motor and anchors PFs within the cell envelope (51, 52). The absence of this structure in the mutant suggests that spatial cues provided by FlhG_Bb_ may influence later stages of motor assembly or stabilization. Thus, FlhG_Bb_ may coordinate multiple levels of flagellar morphogenesis, from the positioning of basal bodies to the assembly of specialized motor structures.

An important remaining question is how FlhG_Bb_ is positioned at the cell poles and division site in *B. burgdorferi*. In several polarly flagellated bacteria, FlhG localization is directed by polar landmark proteins. For example, the scaffold protein HubP anchors FlhG at the old pole in *Vibrio*, thereby spatially restricting flagellar assembly (64). However, no HubP homolog has been identified in *B. burgdorferi*, suggesting that FlhG_Bb_ is recruited through a distinct mechanism. In *Shewanella putrefaciens*, FlhG localization is regulated through an ATP-dependent partner-switching mechanism involving the C-ring protein FliM and the flagellar transcriptional regulator FlrA (also known as FleQ) (60). Interestingly, FlrA shares with approximately 28% sequence identity to the alternative σ^54^ factor RpoN, a master gene regulator of *B. burgdorferi* (65, 66), raising the possibility that a related regulatory pathway may contribute to FlhG_Bb_ positioning. In addition, the conserved N-terminal extension of FlhG functions as a GTPase-activating element for FlhF in several bacteria, coordinating the activities of these two spatial regulators (24, 42). FlhG_Bb_ likewise contains this conserved N-terminal extension, including the characteristic D/NQAxxLR motif, suggesting that reciprocal regulation between FlhG_Bb_ and FlhF may also contribute to its localization. Finally, our biochemical data show that membrane lipids stimulate the ATPase activity of FlhG_Bb_, raising the intriguing possibility that membrane association contributes to its spatiotemporal positioning during the cell cycle. Defining the molecular mechanisms that recruit and position FlhG_Bb_ at the poles and division site will be essential for understanding how *B. burgdorferi* coordinates flagellar biogenesis with polar morphogenesis and cytokinesis. We are currently investigating these mechanisms.

## MATERIALS AND METHODS

### Bacterial strains and growth conditions

A high-passage *Borrelia burgdorferi sensu stricto* strain B31A (wild type) and its isogenic mutants were cultured in Barbour-Stoenner-Kelly II (BSK-II) liquid medium or on semi-solid agar plates at 34°C under 3.4% CO₂, as previously described (13, 67). When required, antibiotics were added for selective pressure at the following concentrations: kanamycin (300 μg/mL), gentamicin (40 μg/mL), or streptomycin (50 μg/mL). *Escherichia coli* NEB 5α (NEB, Carlsbad, CA, USA) was used for DNA cloning and plasmid propagation; M15 (Qiagen, Valencia, CA, USA) was used for recombinant protein expression. For *E. coli* selection, antibiotics were used at the following concentrations: kanamycin (50 μg/mL), spectinomycin (50 μg/mL), or ampicillin (100 μg/mL).

### Preparation of *B. burgdorferi* FlhG (FlhG_Bb_) recombinant protein and antibody

The gene (*BB0269*) encoding the full-length FlhG_Bb_ was PCR amplified using primers P_3_/P_4_ with engineered BamHI and PstI restriction sites at the 5′ and 3′ ends, respectively. The resulting amplicon was cloned into the pGEM-T Easy vector (Promega, Madison, WI) and then subcloned into the pQE80L expression vector (Qiagen, Valencia, CA), which encodes an N-terminal hexa-histidine (His_6_) tag. The recombinant plasmid was transformed into M15 cells for protein expression. FlhG_Bb_ expression was induced with 1 mM isopropyl-β-D-thiogalactoside (IPTG). The His₆-tagged FlhG_Bb_ recombinant protein (His₆-FlhG_Bb_) was purified under denaturing conditions using a nickel-nitrilotriacetic acid (Ni-NTA) agarose column (Qiagen) and subsequently dialyzed overnight at 4°C against 10 mM Tris-HCl buffer. For antibody production, two rats were immunized with 1 mg of purified His_6_-FlhG_Bb_ over a 1-month period, followed by booster injections (100 μg per rat) at weeks 6 and 7. Antiserum was generated by General Bioscience Corporation (Brisbane, CA, USA).

### Site-directed mutagenesis

Site-directed mutagenesis was performed using the Q5 Site-Directed Mutagenesis Kit (NEB, Carlsbad, CA, USA) according to the manufacturer’s instructions. The above constructed FlhG_Bb_ expression plasmid was used as the template. A single amino acid substitution (D69A) was introduced into FlhG_Bb_ using primer pair P_11_/P_12_. The mutation was confirmed by DNA sequencing. The mutated gene was subsequently PCR amplified and subcloned into the pQE80 expression vector (Qiagen) to generate an N-terminal His-tagged fusion protein.

### ATPase hydrolysis assays

Expression vectors encoding His₆-FlhG_Bb_ and its variants were transformed into *E. coli* strain Lemo21 (NEB, Ipswich, MA). Protein expression was induced with 1 mM IPTG for 18 h at 16°C. Cells were harvested for protein purification using Ni-NTA agarose columns under native conditions. Protein concentrations were determined by Bradford assay. ATPase activity was measured by quantifying inorganic phosphate (Pi) release using a colorimetric ATPase/GTPase Activity Assay Kit (Sigma-Aldrich, St. Louis, MO) according to the manufacturer’s protocol. A standard curve generated with the supplied phosphate standard was used to convert absorbance at 620 nm to Pi concentration. For each reaction, 10 µL of purified protein (or assay buffer as a blank control) was mixed with 10 µL ATP and 20 µL assay buffer in a 96-well microplate. After incubation for 30 min at room temperature, 200 µL of colorimetric reagent was added, and the plate was incubated for an additional 30 min to allow color development. Absorbance at 620 nm was measured using a Variskan Lux microplate reader (Thermo Fisher Scientific, Waltham, MA). To measure the potential impact of lipids on the FlhG_Bb_ ATPase activity, a similar experiment was performed with *E. coli* polar lipid (Avanti Research, Birmingham, AL) as previously described (68). For this study, purified His₆-FlhG_Bb_ was preincubated with the lipid vesicle (0.5 mM) for 10 min and then subjected to the kinetic analysis as described below.

For kinetic analysis, 80 nM purified His_6_-FlhG_Bb_ was incubated with increasing ATP concentrations (ranging from 0 to 500 µM as indicated) with or without lipid stimulation. Initial velocities were calculated from Pi production over time, and saturation curves were fit to the Michaelis-Menten equation using GraphPad Prism 10 (GraphPad Software, San Diego, CA, USA). All assays were performed in triplicate in three independent experiments.

### FlhG model preparation and structural comparison

ATP-bound dimeric FlhG_Bb_ (Uniprot ID:Q44911) model was generated using AlphaFold3 (69) (ipTM = 0.88, pTM = 0.90) and compared to AMPPNP bounds *Pseudomonas aeruginosa* FleN (PDB: 5J1J [41]) and *Escherichia coli* MinD (PDB: 3R9I [20]). Structures were visualized and analyzed, and figures were prepared using PyMol.4.

### **Construction of a** ***flhG*** **deletion mutant and its isogenic complemented strains**

The vector for in-frame deletion of *flhG* (*BB0269*) was generated using a PCR-based fusion strategy as previously described (13). Briefly, primer pairs P_5_/P_6_ and P_7_/P_8_ (the latter containing sequences complementary to *aadA1*, a streptomycin resistance marker) were designed to amplify approximately 1-kb regions upstream and downstream of the *flhG* sequence. Three separate PCR reactions were performed to amplify the 5′- and 3′-flanking regions of *flhG* and the *aadA1* cassette (70). The resulting PCR fragments were fused by overlap-extension PCR using primers P_5_/P_8_, generating the deletion construct (*flhG*::aadA1). The resulting construct was transformed into wild-type competent cells by electroporation. Mutant clones were selected on semi-solid agar plates supplemented with streptomycin (50 μg/mL). The resulting mutant clones were confirmed by PCR and DNA sequencing. One mutant clone (∆*flhG*) was chosen for *cis*-complementation through inserting the full-length *flhG_Bb_* gene into an intergenic region between *BB0445* and *BB0446* on the chromosome, as previously described (46, 71). Primer sequences used here are listed in Table S1.

### Measurement of *B. burgdorferi* growth rates

To determine growth kinetics, early stationary-phase cultures of wild-type (WT) B31A, the *flhG* deletion mutant (Δ*flhG*), and the cis-complemented strain (Δ*flhG*^com^) were diluted into fresh BSK-II medium to a final density of 1 × 10⁵ cells/mL and incubated at 34°C. Cell densities were measured daily for up to 8 days using a Petroff–Hausser counting chamber (46). Counts were performed in triplicate for three independent cultures. Data are presented as means ± standard errors of the mean (SEM).

### Bacterial motion tracking analysis and swimming plate assays

The swimming velocity of *B. burgdorferi* cells was quantified using a computer-based motion-tracking system as previously described (8, 12, 72). Briefly, log-phase bacterial cultures were diluted 1:1 in BSK-II medium. A 20-μL aliquot of the diluted culture was mixed with an equal volume of 2% methylcellulose (MC4000; viscosity, 4,000 cP). Cells were recorded using iMovie software on an Apple Mac computer. Videos were exported as QuickTime files and imported into OpenLab (Improvision Inc., Coventry, UK), where frames were cropped, calibrated, and saved as LIFF files. Individual cells were tracked and velocities calculated using Volocity software (Improvision Inc., Coventry, UK). For each strain, at least 20 cells were analyzed for up to 30 s. Swimming plate assays were performed using 0.35% agarose prepared with BSK-II medium diluted 1:10 in Dulbecco’s phosphate-buffered saline (DPBS; pH 7.5), as previously described (12, 67). Swimming ring diameters were measured in millimeters (mm), and mean values were calculated from four independent plates for each strain.

### Gel electrophoresis and Western blot analysis

Sodium dodecyl sulfate-polyacrylamide gel electrophoresis (SDS-PAGE) and immunoblotting using enhanced chemiluminescence (ECL) detection were performed as previously described (13, 50). Briefly, 10–20 μg of total cell lysate was resolved on 12% SDS-PAGE gels and transferred to polyvinylidene difluoride (PVDF) membranes (Bio-Rad Laboratories). Membranes were probed with specific primary antibodies against *B. burgdorferi* proteins, followed by horseradish peroxidase (HRP)-conjugated secondary antibodies and detection using an ECL luminol-based assay. Monoclonal anti-FlaB (H9724) was kindly provided by A. G. Barbour (University of California, Irvine, CA) and monoclonal anti-DnaK by J. Benach (SUNY at Stony Brook, NY). Polyclonal antibodies against FlgG, FliF, FlgE, FlhF, and FliD were described previously (13, 50, 73). Chemiluminescent signals were captured using a ChemiDoc MP imaging system and quantified with Image Lab software (Bio-Rad Laboratories, Hercules, CA). Three independent biological replicates were analyzed for each experiment.

### Construction of plasmids that express FlhG_Bb_-GFP and FliF_Bb_-GFP fusion proteins

These vectors were constructed as previously documented (13). To make the constructs, the full-length *flhG_Bb_* and *fliF_Bb_* genes were PCR amplified with primer sets P_9_/P_10_ and P_13_/P_14_, respectively, in which XhoI and SacII cut sites were engineered. The resultant PCR fragment was first cloned into pGEM-T vector and then subcloned into precut *P_flgB_-flhF_Bb_-gfp*/V2G at the site of XhoI and SacII, yielding the vector of *P_flgB_-flhG_Bb_-gfp*/V2G or *P_flgB_-fliF_Bb_-gfp*/V2G. The final construct was confirmed by DNA sequencing.

### Light and fluorescence microscopy

Cell morphology and motility of *B. burgdorferi* strains were characterized using light microscopy as previously described (13, 74, 75). In brief, *B. burgdorferi* cells in BSK-II medium were videotaped with dark-field illumination at 100× for at least 30 s. Videos were digitized to allow for frame-by-frame observation of cell motion. Fluorescence images were captured using a Zeiss Observer D1 Inverted LED fluorescence microscope at a wavelength of 480 nm using a Zeiss Observer D1 inverted LED fluorescence microscope equipped with an excitation filter (541–569 nm) and an emission filter (581–654 nm). The images were processed using the ZEN Blue software (Zeiss, Germany).

### Fluorescent D-amino acid labeling

HADA (3-[7-hydroxycoumarin]-carboxamide-D-alanine) were synthesized by Tocris following the published protocol (54). Mid-log phase *B. burgdorferi* cultures (2–3 × 10⁷ cells/mL) grown in BSK-II medium were pelleted (9,000 × *g*, 10 min, RT), resuspended in fresh medium, and incubated with 100 µM HADA for 60–120 min at 34°C. Cells were briefly pelleted (9,000 × *g*, 5 min), washed three times with PBS (pH 7.4), and resuspended in PBS for live imaging. Fluorescence images were taken using a Zeiss Observer microscope with a DAPI/FITC filter set. The images were captured and processed using the program ZEN Blue (Zeiss, Germany). For demography analysis, Fiji ImageJ software was used to generate data frame for each cell. At least 400 cells were analyzed per condition. The resulting data were organized into demographs using NumPy (76) and plotted with Matplotlib (77).

### Cryo-electron tomography

*B. burgdorferi* cells in liquid culture were resuspended in PBS to a final OD_600_ of 1.0. A 10-nm gold tracer solution (Aurion) was added, and 5 μL of the mixture was applied to glow-discharged cryo-EM grids (Quantifoil). The grids were blotted from the front with Whatman filter paper and then plunged into a liquid ethane/propane mixture using a GP2 plunger (Leica). The GP2 environmental chamber was maintained at 25°C and 95% humidity, and grids were blotted for 6 s.

Frozen-hydrated specimens were imaged at below −170°C using a Titan Krios G2 300 kV transmission electron microscope (ThermoFisher) equipped with a field emission gun, K3 direct detection camera (Gatan), and GIF BioQuantum K3 Imaging Filter (Gatan). Tilt series were recorded with SerialEM software (78) at 42,000 × magnification, with a physical pixel size of 2.148 Å and a defocus of 4.8 μm. The stage was tilted from −48° to + 48° in 3° increments using a dose-symmetric scheme in the FastTomo script. The total electron dose was ~70 e⁻/Å² distributed over 33 images per tilt series, recorded in dose-symmetric mode.

MotionCor2 (79) was used to correct beam-induced specimen motion during image acquisition. IMOD software was used to create image stacks and then track with fiducial beads to align all images in each tilt series (80, 81). Gctf (82) was used to estimate defocus for all aligned images, and the contrast transfer function (CTF) correction was performed with the ctfphaseflip function in IMOD (83). Tomo3D (84) was then used to generate 8× binned tomograms. In total, 148 tomograms of Δ*flhG* mutant cells, 23 tomograms of Δ*flhG^D69A^* mutant cells*,* 129 tomograms of Δ*flhG* complemented cells were collected (see details in Table S2). The tomograms were segmented using Dragonfly (Version 2022.2, Comet Technologies Canada Inc.) to visualize major cellular features, including the outer membrane, inner membrane, and flagellar filaments of varying lengths.

Protomo (85) was used to determine *in situ* structure of the flagellar motors as described previously (16, 17). Briefly, flagellar motors were manually selected from tomograms, and subtomograms were extracted using 8× binned tomograms for initial alignment and average. The resulting alignment parameters were then applied to subtomograms extracted from 4× binned tomograms for iterative refinement, yielding higher-resolution *in situ* structures of the flagellar motors.

### Statistical analysis

For growth curves, swimming plate assays, and bacterial cell motion tracking analysis, the results are expressed as means ± standard errors of the means (SEM). The significance of the difference between different experimental groups was evaluated using ANOVA (*P* value < 0.01).

## Data Availability

*In situ* flagellar motor structure derived from cryo-ET and subtomogram averaging has been deposited in the Electron Microscopy Data Bank (EMDB) as Δ*flhG* (EMD-76329).

## SUPPLEMENTAL MATERIAL

Supplemental tables and figures mbio.01834-26-s0001.pdf Two tables and six figures.

Video S1 mbio.01834-26-s0002.mp4 Tracking analysis of WT.

Video S2 mbio.01834-26-s0003.mp4 Tracking analysis of *flhG* mutant.

Video S3 mbio.01834-26-s0004.mp4 Tracking analysis of *flhG* complemented strain.

Video S4 mbio.01834-26-s0005.mp4 Tracking analysis of FlhG D69A mutant.

ASM does not own the copyrights to Supplemental Material that may be linked to, or accessed through, an article. The authors have granted ASM a non-exclusive, world-wide license to publish the Supplemental Material files. Please contact the corresponding author directly for reuse.
