# Supplementary material for: MinD-like ATPase FlhG synchronizes flagellation and cell division in spirochetes
Source: mBio. 2026 Sep 24;17(10):e01834-26. doi: 10.1128/mbio.01834-26 (PMC13712777; doi:10.1128/mbio.01834-26)
Supplement: Supplementary file 1 [file mbio.01834-26-s0001.pdf]

Supplementary Data

Table S1. Oligonucleotide primers used in this study

Primers	Sequences (5'-3')	Note [a]
P1	ATGAGGGAAG CGGTGATCGC CGAAG	*aadA1* cassette, deletion of *flhG* [F]
P2	TTATTTGCCGACTACCTTGGTGATC	*aadA1* cassette, deletion of *flhG* [R]
P3	GGATCCATGGAAGATCAAGCTCAAAG	FlhG recombinant protein [F]
P4	CTGCAG TTATTCCACACCAAAAAATC	FlhG recombinant protein [R]
P5	TGAAAGGG TTAGAGAGGATG	5'-flanking region of *flhG*, deletion of *flhG* [F]
P6	CTTCGGCGATCACCGCTTCCCTCAT TATAACTTTCCTTAATAATAAG	5'-flanking region of *flhG*, deletion of *flhG* [R]
P7	GATCACCAAGGTAGTCGGCAAATAA GACATTATGTTTATAAGCAG	3'-flanking region of *flhG*, deletion of *flhG* [F]
P8	TTTATAAGGTACCGAGAGCT	3'-flanking region of *flhG*, deletion of *flhG* [R]
P9	CTCGAGATGGAAGATCAAGCTCAAAG	FlhG-GFP fusion [F]
P10	CCGCGG TTCCACACCAAAAAATC	FlhG-GFP fusion [R]
P11	GCTATTGGAATGGCTAATGT	*flhG* site-directed mutagenesis [F]
P12	AGCATCAAGT ATTAACACCT	*flhG* site-directed mutagenesis [R]
P13	CTCGAG TTGAGCAAATTTTTTACTAAT	FliF-GFP fusion [F]
P14	CCGCGG CGCGTTTTTC AAAAGCCATG	FliF-GFP fusion [R]

[a] The underlined nucleotides are the engineered restriction cut sites for DNA cloning; [F] forward; [R] reverse.

Table S2. Summary of cryo-ET data collection.

	Δ*flhG*	D69A	Δ*flhG*^{com}
# of tomograms	148	23	129
# of motors	485	130	1004
N/N (motors/cell)	~3	~6	~8
Magnification	42k	42k	42k
Pixel size	2.14 Å/pixel	2.14 Å/pixel	2.14 Å/pixel
Defocus	-4.8 μm	-4.8 μm	-4.8 μm

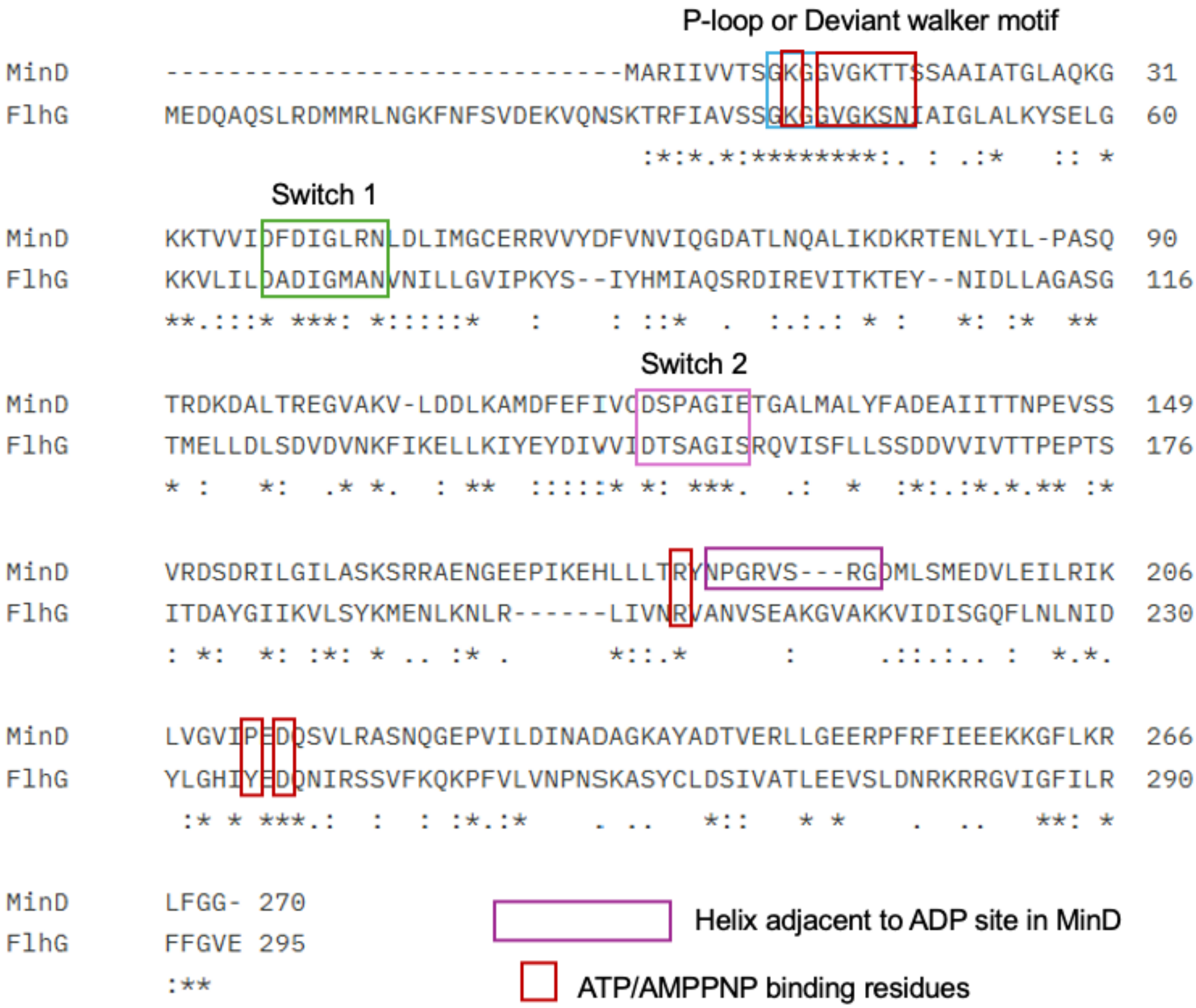

Figure S1. Sequence comparison of *B. burgdorferi* FlhG (BB0269) and *E. coli* MinD.

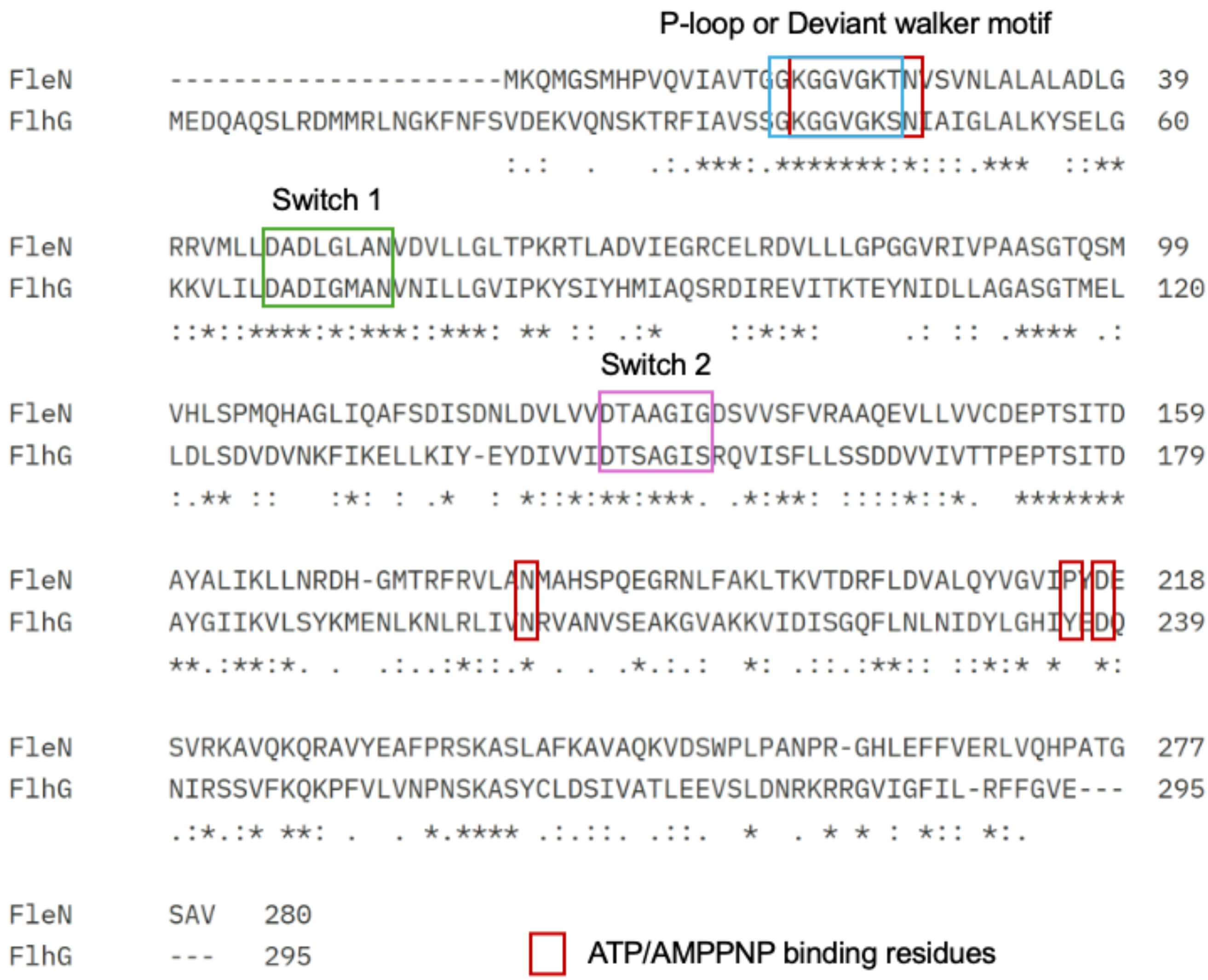

Figure S2. Sequence comparison of *B. burgdorferi* FlhG (BB0269) and *P. aeruginosa*.

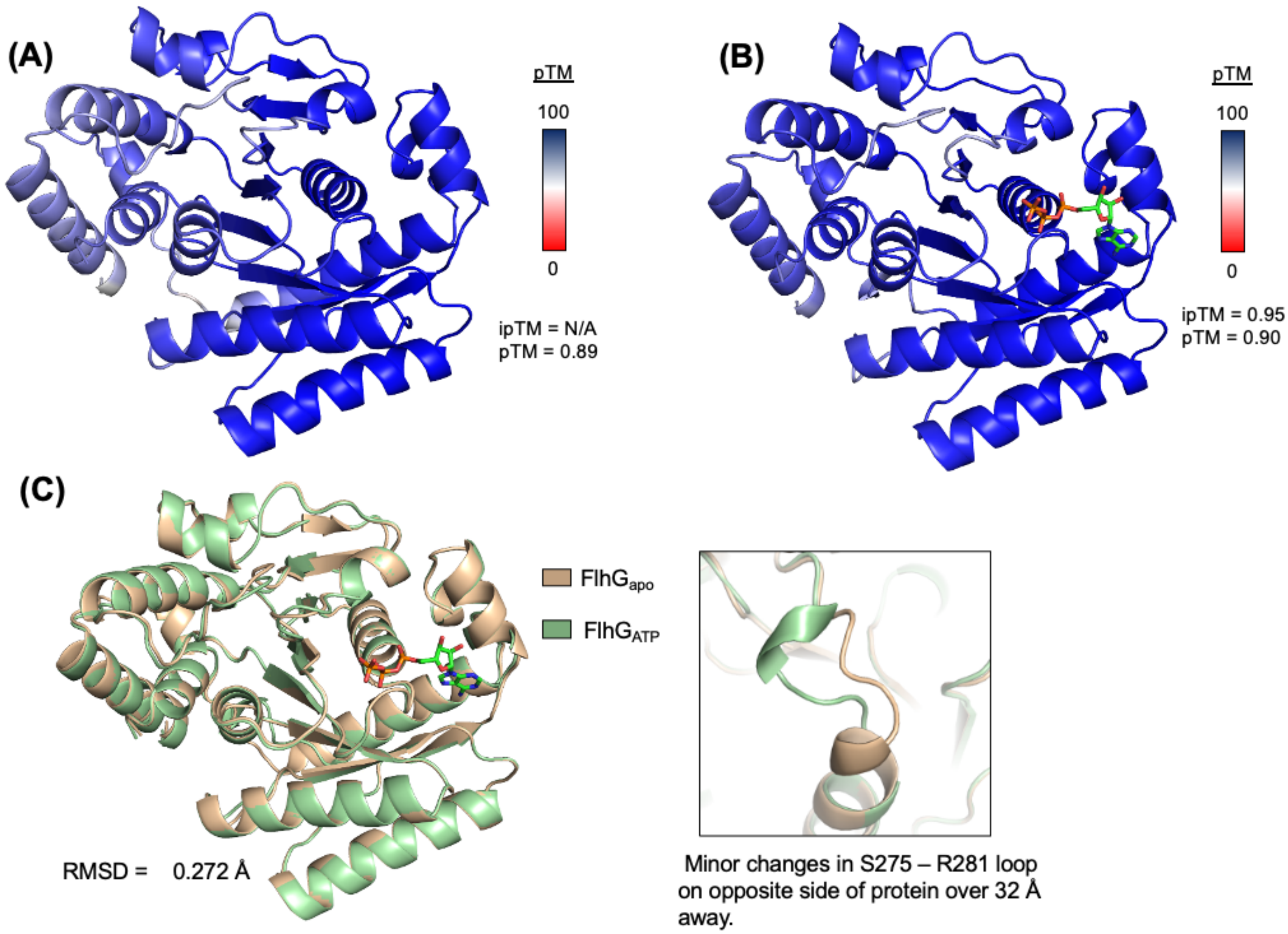

Figure S3. AlphaFold structure of FlhG$_{Bb}$ monomer with or without ATP.

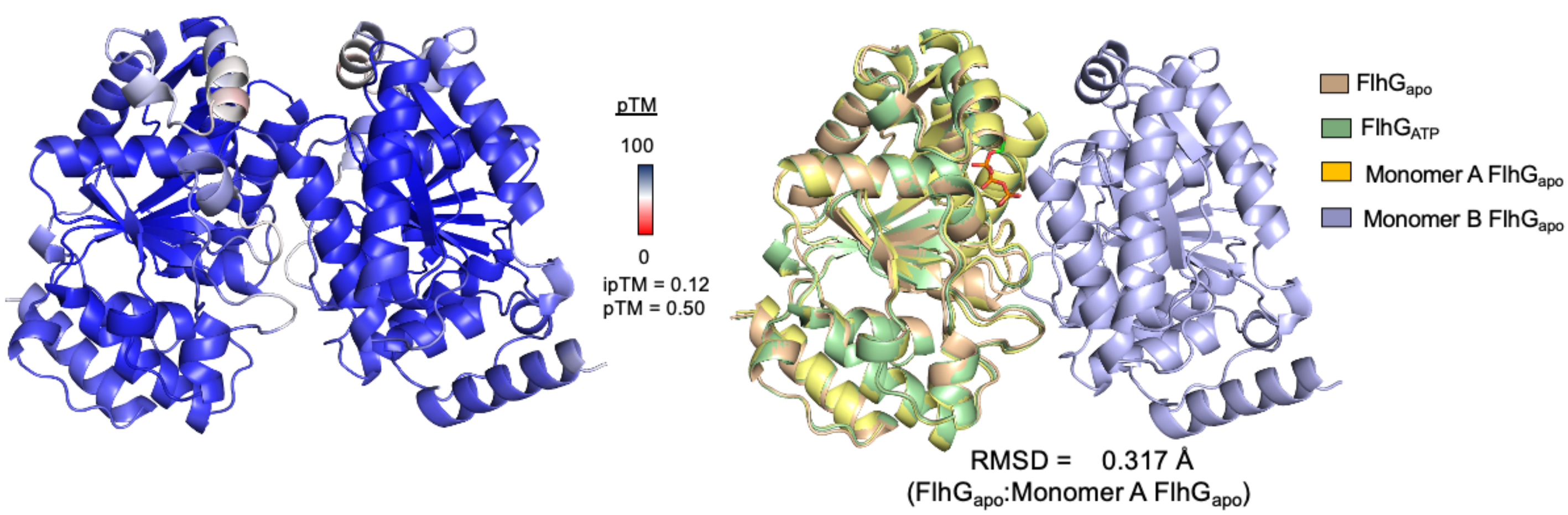

Figure S4. AlphaFold structure of FlhG$_{Bb}$ dimer with or without ATP.

```
B.subtilis          MNRYDQAATLRAKMEKR----ERVLPMVYSQKAKTLAVISGKGGVGKSNITLNMALALQD     56
C.jejuni            --MNNQANKLRNLMSQNGTK------KS--QNTHFIAITSGKGGVGKSTISANLANVLAN     50
H.pylori            --MNNQASRLDNLMNIKNPKSFFD--NK--GNTKFIAITSGKGGVGKSNISANLAYSLYK     54
B.burgdorferi       --MEDQAQSLRDMMRLNGKFNFSVDEKVQNSKTRFIAVSSGKGGVGKSNIAIGLALKYSE     58
S.putrefaciens      ------------MTLDQASGLRMMNQPYNEKVKVIAVTGGKGGVGKTSVSINTAVALAE     47
V.cholera           ---------MTNKMIYDQASGLRRLTQPSV--TKVISVTGGKGGVGKSNVTLGMAICMAK     49
                                 *                  .: ::: .*******:.:: . *    .
```

Figure S5. Sequence alignment of FlhG N-termini from six different bacteria.

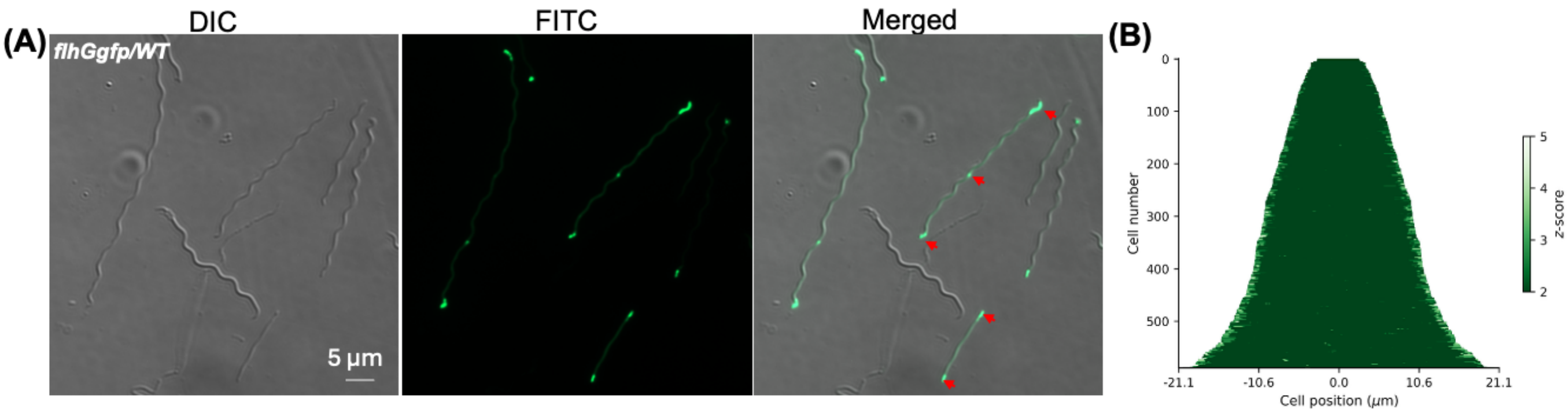

Figure S6. (A) Localization of FlhG-GFP in *B. burgdorferi* wild type (WT). Red arrows point to the cellular localization of FlhG-GFP at cell poles and midcell. **(B)** Demographic distribution of FlhG-GFP in *B. burgdorferi* wild type cells (n= 590 cells).
